# Laparoscopic donor nephrectomy: an increasingly common cause for testicular pain and swelling

**DOI:** 10.1308/003588412X13171221592177

**Published:** 2012-09

**Authors:** M Jalali, S Rahmani, AD Joyce, JJ Cartledge, MH Lewis, N Ahmad

**Affiliations:** ^1^Leeds Teaching Hospitals NHS Trust,UK; ^2^Cwm Taf Health Board,UK

**Keywords:** Laparoscopic donor nephrectomy, Live donor, Open donor nephrectomy, Renal transplant, Testicular pain/swelling

## Abstract

**INTRODUCTION:**

Laparoscopic donor nephrectomy (LDN) is now a well established method for kidney procurement from living donors. In our centre, LDN is currently offered only to donors suitable for a left nephrectomy. The aim of this study was to investigate the incidence of testicular pain and swelling following LDN.

**METHODS:**

A total of 25 left-sided LDN male patients were assessed in a prospective structured interview together with a control cohort of 25 male patients who had undergone left-sided open donor nephrectomy (ODN).

**RESULTS:**

Data were collected on testicular pain, swelling, numbness, urinary symptoms and sexual dysfunction from all 50 patients (100% response rate). Of the 25 LDN patients, 11 (44%) experienced ipsilateral testicular pain and/or swelling. In most instances, pain was of immediate onset, mild to moderate in severity, lasted for a few days to several weeks and was associated with testicular swelling (10 of 11 cases). However, testicular pain and/or swelling were not apparent in ODN patients, with only 2 of 25 (8%) experiencing mild testicular pain, 1 of whom also had swelling.

**CONCLUSIONS:**

Testicular pain and swelling following LDN is a common problem. It is underreported in the literature and should be included in the differential diagnoses of testicular pain and swelling. Further investigation is required to confirm our findings.

A large number of men are referred with testicular pain and/or discomfort to general surgical and urological outpatient clinics. Although the differential diagnoses of testicular neuralgia include testicular trauma, carcinoma, torsion and infection, many such patients have no definite cause identified and are reassured accordingly.

The number of patients requiring renal replacement therapy is increasing steadily worldwide. While transplant remains the best treatment for renal failure, the supply of donor organs has remained constant over the last decade. In order to redress this imbalance, over the last few years there has been rapid expansion in the use of live donor renal transplantation in Europe and North America.

The advent of laparoscopic donor nephrectomy (LDN), first described over 15 years ago,^1^ has been a vital development in the field of renal transplantation and may have helped increase donation rates in this area. By affording the benefits of minimal access surgery to healthy donors, LDN has transformed the process of live renal transplantation and become a more attractive alternative to open donor nephrectomy (ODN). LDN is not only associated with equivalent graft function and survival compared with ODN but it has also been shown to be associated with better post-operative recovery and aesthetic outcome.^2–5^

We report our experience of patients undergoing LDN, a now well established method for the procurement of kidneys, and donors’ post-operative symptoms including testicular pain and swelling. In our early experience of LDN, several patients have complained of significant testicular pain and swelling. We therefore reviewed our LDN patient group specifically to look at the incidence and severity of testicular pain and swelling post-operatively, and in doing so endeavoured to shed more light on this underreported but clinically significant problem. We investigated whether testicular pain and swelling are indeed more common following LDN and whether there was a causal link between LDN and symptoms of testicular pain and swelling.

## Methods

A total of 54 left-sided LDNs were undertaken over the study period from February 2005, 25 of which were in male patients. For anatomical reasons, all were harvested on the left side using a totally transperitoneal laparoscopic approach. All procedures were undertaken by one very experienced laparoscopic surgeon, without any learning curve required. The maximum intra-abdominal pressure was 12–15mmHg. The left colon was mobilised medially from the splenic flexure to the pelvic brim. Each kidney was retrieved intact with an envelope of Gerota's fascia. The gonadal vein was divided early at mid-ureter level and the ureter was divided late, immediately prior to vascular stapling. The ureteric pedicle was used for retraction to facilitate dissection. The renal vein was divided medial to the entrance of the gonadal and adrenal veins. Each kidney was retrieved in a bag through an 8cm skin creased incision at the left iliac fossa.

An equal number of consecutive left-sided ODN male donors from the same unit was used as a control cohort. All 25 ODNs in the control cohort were performed using a mini open nephrectomy approach with an 8–10cm anterior incision extending from the tip of the 12th rib. Gerota’s envelope was dissected off the kidney prior to its delivery, and both the adrenal and gonadal veins were divided at their junction with the renal vein. The ureter was divided early and the renal vein divided medial to the entrance of the gonadal and adrenal veins as in the LDNs. The ureteric pedicle was not used for retraction at any stage, with minimal dissection of the retroperitoneum along the length of the ureter.

Patient demographics were collected from a prospectively maintained database kept in the department. All 50 patients were interviewed post-operatively with an identical patient proforma (Table 1). Data were collected on testicular pain, swelling and numbness, as urinary symptoms and sexual dysfunction. Pain severity was assessed using a visual analogue scale. Statistical analysis of data was carried out using SPSS® (SPSS, Chicago, IL, US).

**Table 1:** Areas of enquiry during the structured interview

1. Pain in the left testicle or scrotum
2. Pain severity on a visual analogue scale
3. Swelling of the left testicle or scrotum
4. Numbness in the area of the left scrotum or groin
5. Erectile dysfunction
6. Premature/delayed ejaculation
7. Pain during intercourse
8. Blood in semen
9. Previous testicular pain or swelling or treatment
10. Previous groin surgery for hernia or testicular/scrotal surgery
11. Frequency/urgency/nocturia/weak urine flow/intermittent urine flow/strain to void/incomplete emptying

## Results

Overall, information from 25 LDNs and 25 ODNs was analysed. The mean donor ages in the LDN and ODN cohorts at the time of surgery were 41 years (standard deviation [SD]: 7 years) and 42 years (SD: 11 years) respectively. Data were collected from all patients in the LDN and ODN groups (100% response rate).

Of the 25 LDN patients, 11 (44%) experienced testicular pain and/or swelling. One of the eleven patients experienced testicular swelling alone for seven weeks. Pain was ipsilateral (left) in all 25 cases, occurring immediately after the operation and lasting for up to four weeks. In 4 of the 10 patients with testicular pain, the pain had persisted for more than 3 years. The mean pain score was 4.6 (SD: 2.0). Five of the ten individuals who experienced new onset post-operative ipsilateral testicular pain had a past history of vasectomy and/or inguinal hernia repair surgery.

Swelling typically lasted from a few days to several weeks after the operation. The mean duration of resolved testicular swelling was 8 days (SD: 6 days). However, 6 of 10 patients had persistent testicular/scrotal swelling that had remained since the LDN procedure. There was no past history of scrotal swelling or pain in these individuals.

Testicular pain and swelling following LDN was not associated with any urinary symptoms or sexual dysfunction. There was no recorded dysuria, incontinence, retention, erectile dysfunction or fertility problems. LDN patients did not give any history of numbness or paraesthesia in the scrotum or inguinal region.

All of the 25 ODN patients received a left-sided ODN. Testicular pain and swelling were not apparent, with only two patients experiencing ipsilateral mild testicular pain. In one case, pain occurred immediately after the operation and resolved within two weeks of surgery. The other case was that of immediate post-operative testicular pain and swelling that had persisted since the ODN procedure (Table 2).

**Table 2:** Testicular pain and swelling in laparoscopic and open donor nephrectomy patients

	**LDN** (*n*=25)	**ODN** (*n*=25)
Mean donor age	41 (SD: 7)	42 (SD: 11)
Testicular pain alone	0	1
Testicular pain and swelling	10	1
Testicular swelling alone	1	0
Mean visual analogue scale score	4.6 (SD: 2.0)	3.5 (SD: 0.7)

LDN = laparoscopic donor nephrectomy; ODN = open donor nephrectomy; SD = standard deviation

## Discussion

Since the introduction of LDN at our institution, approximately a third of all nephrectomies are performed using this technique. At present, LDN is offered only to donors suitable for left nephrectomy in view of the longer left renal vein. It is our intention to perform LDN for right kidney procurement in the near future.

Our standard practice with LDN is to divide the gonadal vein early up to the mid-ureter level. The ureter is then divided late, immediately prior to vascular stapling, and the ureteric pedicle is used for retraction to facilitate renal dissection. This technique may well contribute to the subsequent testicular neuralgia following LDN.

It has been our experience that a considerable number of male donors who have received LDN complain of testicular pain and swelling following their operation. This investigation was undertaken in an attempt to elucidate whether testicular pain and swelling were more common for LDN patients and also whether there was a causal link between LDN and these symptoms.

A study in 2003 by Kim *et al* noted that 10% of their 145 male patients undergoing left-sided LDN complained of testicular pain.^6^ A year later, Su *et al* demonstrated a 1% incidence of testicular pain in their LDN cohort of 381 patients, the vast majority (95%) of whom had received left-sided procedures.^7^ The sex ratio in this study was not reported and it is therefore not possible to define the exact incidence of cases with testicular pain. In 2005 Brook *et al* showed a 3% incidence of ipsilateral testicular pain in 70 patients treated at their centre following LDN,^8^ and in 2008 Gjertson and Sundaram reported that 55% of 20 LDN patients had developed ipsilateral testicular pain.^9^

Other plausible explanations as to the cause of testicular pain have been suggested by Kim *et al*,^6^ attributing this pain to testicular neural connections and their division during dissection of the periureteral tissue or transection of the spermatic cord.^6^ In 2005 Burgos *et al* found a significant reduction in renal blood flow and function when employing LDN in pigs.^10^ This would support the theory, suggesting that orchialgia can be attributed to testicular venous congestion due to reduced renal venous drainage. In a later study, Burgos *et al* also demonstrated that reduction in renal blood flow during LDN could be avoided by adequate peri-operative intravascular volume expansion, which in turn may reduce the incidence of orchialgia post-operatively.^11^ In a further study, Gjertson and Sundaram concluded that harvesting the gonadal vein as well as the obstruction of lymphatics from the scrotal region, may further account for the incidence of post-operative ipsilateral testicular pain.^9^

We believe that division of the gonadal vein at the mid-ureter and retraction of the ureteric pedicle during dissection in LDN may contribute to post-operative testicular pain and swelling. Furthermore, extensive mobilisation of the left colon may damage the neural plexus supplying the testis and may also disrupt the lymphatic drainage.

We have found no reports of ipsilateral testicular pain or swelling following right-sided LDN. This may be attributed to the fact that in right-sided LDN, the gonadal vein is not divided and less extensive mobilisation of the right colon is required, thereby preserving the neural plexus and lymphatic drainage. Similarly, testicular pain and swelling is not frequently reported in laparoscopic nephrectomies of either side for other aetiologies. In such cases, the gonadal vein is not divided and the renal vein is divided lateral to the insertion of the gonadal vein. In addition, the ureter is divided early during the procedure, thus avoiding the retraction of the ureteric pedicle for the duration of surgery as in LDN.

Our study has demonstrated that approximately half of patients undergoing LDN for donor renal transplantation experience significant ipsilateral testicular pain. Most complain of associated scrotal swelling and in a notable proportion of these patients pain and swelling last for several weeks and sometimes up to several years. The pain has been sufficient to compromise a return to activities of daily living and consequently has a major impact on post-operative quality of life. The improved post-operative recovery intended with the advent of laparoscopic surgery would therefore be diminished in these patients. The fact that some patients are still experiencing testicular pain in excess of a year post-operatively remains to be confirmed in further studies and, if confirmed, may even be a contraindication to LDN in the future.

Further investigation may also attempt to define the incidence of post-operative testicular pain and swelling following LDN for other indications such as benign disease, chronic infection and trauma. To our knowledge, these symptoms have not been reported as a complication of LDN for indications other than donor renal transplant^6–8^ and oncological renal surgery.^9^ We have adapted our surgical practice in an attempt to prevent these complications, by preservation of the gonadal vein until late in the procedure and avoidance of damage of the neurovascular bundle below the point of the gonadal vein ligation at the renal vein. In order to reduce damage to the sensory nerves of the testicle in the periureteral tissue, careful dissection of the ureter is also undertaken. We aim to reaudit our results after implementing these changes when we have obtained a sufficient number of patients in both open and laparoscopic groups.

## Conclusions

In general surgical and urological clinics, testicular pain and swelling are frequently observed presenting symptoms with numerous differential diagnoses. Since it is now becoming a widely performed procedure, LDN must be borne in mind as an increasingly common cause for such symptoms.
